# RGS10 Negatively Regulates Platelet Activation and Thrombogenesis

**DOI:** 10.1371/journal.pone.0165984

**Published:** 2016-11-09

**Authors:** Nicole R. Hensch, Zubair A. Karim, Kirk M. Druey, Malú G. Tansey, Fadi T. Khasawneh

**Affiliations:** 1 Department of Pharmaceutical Sciences, College of Pharmacy, Western University of Health Sciences, Pomona, CA 91766, United States of America; 2 Molecular Signal Transduction Section, Laboratory of Allergic Diseases, NIAID/NIH, 10 Center Drive Room 11S244B, Bethesda, Maryland 20892, United States of America; 3 Department of Physiology, Emory University School of Medicine, Atlanta, GA 30322, United States of America; Ludwig-Maximilians-Universitat Munchen, GERMANY

## Abstract

Regulators of G protein signaling (RGS) proteins act as GTPase activating proteins to negatively regulate G protein-coupled receptor (GPCR) signaling. Although several RGS proteins including RGS2, RGS16, RGS10, and RGS18 are expressed in human and mouse platelets, the respective unique function(s) of each have not been fully delineated. RGS10 is a member of the D/R12 subfamily of RGS proteins and is expressed in microglia, macrophages, megakaryocytes, and platelets. We used a genetic approach to examine the role(s) of RGS10 in platelet activation *in vitro* and hemostasis and thrombosis *in vivo*. GPCR-induced aggregation, secretion, and integrin activation was much more pronounced in platelets from *Rgs10*^***-/-***^ mice relative to wild type (WT). Accordingly, these mice had markedly reduced bleeding times and were more susceptible to vascular injury-associated thrombus formation than control mice. These findings suggest a unique, non-redundant role of RGS10 in modulating the hemostatic and thrombotic functions of platelets in mice. RGS10 thus represents a potential therapeutic target to control platelet activity and/or hypercoagulable states.

## Introduction

Platelets have an integral role in maintaining hemostasis., Excessive platelet activation promotes debilitating pathophysiology including heart attacks and strokes. The majority of bloodborne factors activating platelets (e.g. thrombin, ADP, thromboxane A_2_ and epinephrine) utilize G-Protein Coupled Receptors (GPCRs) to initiate physiological responses such as aggregation and secretion [1]. GPCRs on the platelet surface induce G protein (GP) activation through the exchange of guanosine diphosphate (GDP) for guanosine triphosphate (GTP), resulting in a host of cellular responses: calcium release (i.e., G_q_), inhibition of adenylyl cyclase (i.e., Gi), and activation of Rho GTPase (i.e., G_13_), amongst others. In contrast, prostaglandin I_2_ (PGI_2_)-mediated activation of adenylyl cyclase through Gs leads to generation of cyclic adenosine monophosphate (cAMP), which inhibits platelet activation. Regardless of the specific pathway, Gα uniformly terminates signaling by hydrolyzing GTP and returning to the inactive, GDP-bound state. Although soluble inducers of platelet activation have been well characterized, platelet-intrinsic factors controlling reactivity to humoral mediators have not been fully delineated.

Regulators of G protein signaling (RGS) proteins negatively regulate GPCRs by acting as GTPase activating proteins (GAPs) and thereby augmenting GP cycling back to the inactive form [2]. More than 30 RGS proteins have been identified by the presence of a conserved RGS domain that mediates binding to Gi, Gq, and/or G12/13 (but not Gs) and GAP activity[3]. Rodent and human platelets express several RGS proteins including RGS2, RGS10, RGS16, and RGS18 [4–7]. Mice genetically modified to express Giα2 containing an RGS-insensitive mutation (either globally or limited to hematopoietic cells) exhibited markedly increased platelet aggregation at sites of blood vessel injury, suggesting the importance of RGS-Gα interactions for platelet functions [8]. In patients with metabolic syndrome and aspirin resistant platelets, expression of RGS2, 10, and 18 was significantly increased compared to aspirin sensitive platelets [9]. These studies provide both direct and indirect evidence that RGS proteins are physiologically relevant regulators of platelet reactivity and hemostatic functions.

Here we studied platelet development and platelet functions in *Rgs10*^***-/-***^ mice. We found that platelets isolated from this strain displayed significantly increased aggregation, secretion, and integrin activation compared to those from WT littermates. We observed that *Rgs10*^***-/-***^ mice exhibited shortened tail bleeding times and occlusion times in a FeCl_3_-induced injury model of thrombus formation. Together, our findings support the hypothesis that RGS10 plays a critical role in platelet-mediated hemostasis and thrombogenesis.

## Materials and Methods

### Reagents and materials

Collagen, ADP, thrombin, stir bars, and other disposables were from ChronoLog (Havertown, PA). U46619 and PGI_2_ was obtained from Cayman Chemical Company (Ann Arbor, MI). Apyrase was purchased from Sigma Aldrich (St. Louis, MO). PAR4 agonist peptide (TRAP4) was from Peptides International (Louisville, KY). The CD62P antibody was obtained from BD Biosciences (San Jose, CA). PE-conjugated rat anti-mouse Integrin αIIbβ3 (active form) JonA antibody was purchased from Emfret Analytics (Eibelstadt, Germany). RGS16 and RGS18 antibodies used for protein detection from western blot were obtained from Santa Cruz Biotechnology (Santa Cruz, CA). RGS10 antibodies used for protein detection were purchased from Santa Cruz Biotechnology (Santa Cruz, CA) and Abcam (Cambridge, MA). Other reagents were of analytical grade.

### Animals

*Rgs10*^*-/-*^ mice were generated as described before [10] and genotyped using a PCR-based method. PCR was performed using following primers: R10GenF: 50 -CCACGAGGAAGTGAAGTGAAAGCTTT-30, R10GenR 50 -AGTCAGTTCTGAGTGTGTGAAAGTGC-30, and LTR2: 50 –AAATGGCGTTACTTAAGCTAGCTTGC-30 with the following PCR condition: denaturation: 94 extension for 72 ^o^C for 10 min. DNA were run in 1% agarose gel and visualized in the gel documentation system. The following products were observed: WT: 400 bp, Het: 400/200 bp, and Mutant: 200 bp. Western blotting was performed in order to compare WT and *Rgs10*^***-/-***^ platelets with regards to RGS10, RGS16, and RGS18 expression, in a 12% SDS-PAGE gel. Mice were housed in groups of 1–4 at 24 ^o^C, under 12/12 light/dark cycles, with access to water and food ad libitum. All experiments involving animals were performed in compliance with the institutional guidelines, and were approved by the Western University of Health Sciences Institutional Animal Care and Use Committee.

### Platelets preparation

Platelets were prepared as previously described [11]. Mouse blood was collected from a ventricle and the citrated (0.38%) blood was mixed with phosphate-buffered saline, pH 7.4, and was incubated with PGI_2_ (10 ng/mL; 5 min), followed by centrifugation at 237x g for 10 min at room temperature (RT). Platelet-rich plasma (PRP) was recovered and platelets were pelleted at 483x g for 10 min at RT. The pellets were resuspended in HEPES/Tyrode buffer (20 mM HEPES/KOH, pH 6.5, 128 mM NaCl, 2.8 mM KCl, 1 mM MgCl_2_, 0.4 mM NaH_2_PO_4_, 12 mM NaHCO_3_, 5 mM D-glucose) supplemented with 1 mM EGTA, 0.37 U/mL apyrase, and 10 ng/mL PGI_2_. Platelets were then washed and resuspended in HEPES/Tyrodes (pH 7.4) without EGTA, apyrase, or PGI_2_. Platelets were counted with an automated hematology analyzer (Drew Scientific Dallas, TX) and adjusted to the indicated concentrations.

### *In vitro* platelet aggregation

The PRP from *Rgs10*^**-/-**^ and WT mice was stimulated with 1–2.5 μM ADP, 40–80 μM TRAP4, 0.25–0.5 μM U46619 and 0.1–0.2 U/ml thrombin. Platelet aggregation was measured by the turbidometric method using models 490 or 700 aggregometry systems (Chrono-Log Corporation, Havertown, PA). Each experiment was repeated at least 3 times and blood was pooled from at least three separate groups of eight mice.

### Flow cytometric analysis

The PRP from *Rgs10*^**-/-**^ and WT was stimulated with the 5 μM ADP, and 80 μM TRAP4 for 5 minutes at room temperature. The reaction was stopped by fixing cells with 4% paraformaldehyde in PBS. Fixed platelets were labeled with PE-conjugated rat anti-mouse Integrin αIIbβ3 (active form) JonA antibody or CD62P antibody. Samples (10^5^ platelets/100 μl) were then analyzed using an ACURI C6 flow cytometer as previously described [12].

### Tail bleeding time

The *Rgs10*^***-/-***^ and WT mice were used for the tail bleeding assay. Hemostasis was examined using the tail transection technique [13–16]. Briefly, mice were anesthetized with isoflurane and place on a 37 ^o^C homeothermic blanket and their tails were transected 5 mm from the tip. The tail was placed in saline at 37 ^o^C and the time to bleeding cessation was measured. Bleeding stoppage was not considered complete until bleeding had stopped for 1 min. When required, measurements were terminated at 15 min.

### *In vivo* FeCl_3_ injury thrombosis model

These studies were performed as described previously [13, 15, 16]. Briefly, *Rgs10*^***-/-***^ and WT mice (8–10 weeks old) were anesthetized with isoflurane. The left carotid artery was exposed and cleaned, and baseline carotid artery blood flow was measured with Transonic micro-flowprobe (0.5 mm, Transonic Systems Inc., Ithaca, NY). After stabilization of blood flow, 7.5% ferric chloride (FeCl_3_) was applied to a filter paper disc (1-mm diameter) that was immediately placed on top of the artery for 3 min. Blood flow was continuously monitored for 45 min, or until blood flow reached stable occlusion (zero blood flow for 2 min). Time to vessel occlusion was calculated as the difference in time between stable occlusion and removal of the filter paper (with FeCl_3_). An occlusion time of 10 min was considered as the cut-off time for the purpose of statistical analysis.

### Statistical analysis

All experiments were performed at least three times. Analysis of the data was performed using GraphPad PRISM statistical software (San Diego, CA) and presented as mean ± SEM. The Mann-Whitney test was used for the evaluation of differences in mean occlusion and bleeding times. Analysis was also conducted using unpaired t-test for FACS analysis, and similar results were obtained with this test for the previous differences. Significance was accepted at P < 0.05 (two-tailed P value), unless stated otherwise.

## Results

### RGS10 deficiency does not affect platelet RGS protein levels, or peripheral blood count

**Fig 1:**
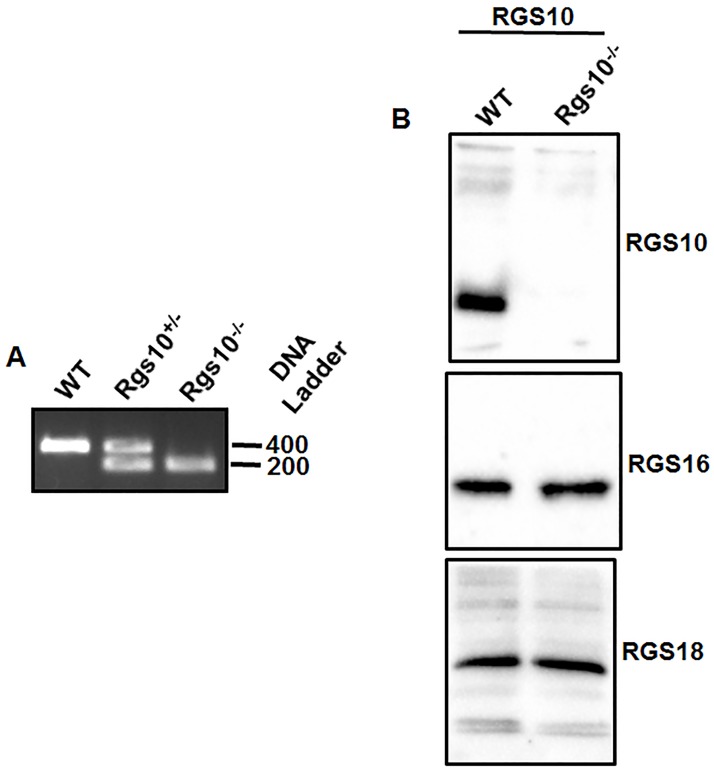
RGS10 deficiency does not affect platelet RGS protein levels. (A) DNA was isolated, and PCR was performed as described in the “Methods” section. DNA was separated on a 1% agarose gel and visualized using a gel documentation system. (B) Platelet extracts (2 x 10^8^/ml) were prepared from wild type (WT) and the *Rgs10*^***-/-***^ mice. Proteins were probed using Western Blot.

To discern the direct role of RGS10 in platelets, we studied platelet development and function in mice containing a global knockout of the *Rgs10* gene (*Rgs10*^*-/-*^). The RGS10 knockout strain showed no physical differences in comparison to their wild-type counterparts, with both strains producing viable, and healthy offspring. We verified the genotype of the RGS10 mice by PCR analysis of genomic DNA (Fig 1A). RGS10 protein was readily detected in lysates of purified platelets from WT mice whereas those from *Rgs10*^*-/-*^ mice lacked expression of RGS10 as expected. RGS10 deficiency did not affect expression of other RGS proteins expressed in platelets (RGS16 and RGS18) (Fig 1B). Furthermore, analysis of peripheral blood samples of *Rgs10*^*-/-*^ mice and littermates for their hematologic profile showed that *Rgs10*^*-/-*^ mice have normal platelet counts, including mean platelet volume (MPV) with respect to WT mice (Table 1). All other blood parameters analyzed, including red blood cells and total white blood cells, were also normal in *Rgs10*^*-/-*^ mice. These data suggest that RGS10 deficiency does not affect the expression levels of other platelet RGS proteins or thrombopoiesis.

**Table 1:** Peripheral blood cell counts in WT and *Rgs10*^*-/-*^ mice.

	WT	*Rgs10*^*-/-*^	P values
**Platelets**	1107.32 ± 3.2	1110.29 ± 2.9	NS
**MPV**	4.75 ± 2.75	4.92 ± 3.02	NS
**Red Blood Cells**	8.26 ± 1.25	9.01 ± 1.54	NS
**Lymphocytes**	6.21 ± 1.65	6.75 ± 1.45	NS
**Monocytes**	0.39 ± 1.32	0.41 ± 1.69	NS
**Granulocytes**	2.45 ± 2.05	2.39 ± 2.78	NS

Blood was collected from the heart and was counted as mentioned in Methods section. All counts are thousands per microliter, except for red blood cells which are millions per microliter. NS: not significant. Data are represented as SD.

### Deletion of RGS10 alters agonist-induced platelet activation

**Fig 2:**
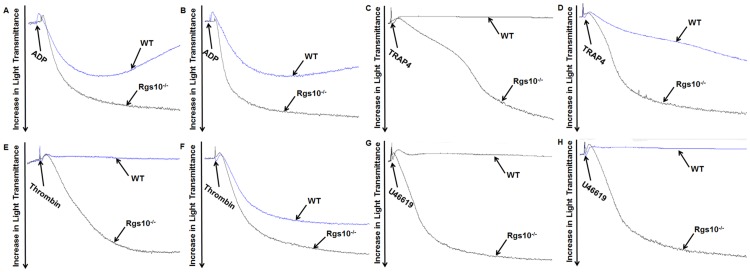
Deletion of RGS10 alters agonist-induced platelet activation. Platelets from *Rgs10*^***-/-***^ or WT mice (2 x 10^8^/ml) were exposed to (A) 1 μM ADP, (B) 2.5 μM ADP, (C) 40 μM TRAP4, (D) 80 μM TRAP4, (E) 0.1 U/ml thrombin, (F) 0.2 U/ml thrombin, (G) 0.25 μM U46619, and (H) 0.5 μM U46619. Platelet aggregation was measured using constant stirring. Each experiment was repeated at least 3 times, with blood pooled from a group of eight mice.

We first examined how platelet activation is affected by *Rgs10* gene deletion by analyzing aggregation following treatment with GPCR agonists including ADP, a peptide ligand of the protease activated receptor PAR4 (TRAP4), and the thromboxane receptor agonist, U46619, which is measured as a decrease in turbidity of a platelet-containing solution over time. Platelets from *Rgs10*^**-/-**^ mice did not exhibit spontaneous aggregation, but rather aggregated significantly more than platelets from WT mice in response to several concentrations of ADP (Fig 2A and 2B), the PAR4 ligand (Fig 2C and 2D), thrombin (Fig 2E and 2F), and U46619 (Fig 2G and 2H). Due to the lack of a response to lower concentrations of agonists, we were unable to fully determine the effect of RGS10 deficiency on agonist efficacy and potency (i.e. EC_50_, E_max_).

**Fig 3:**
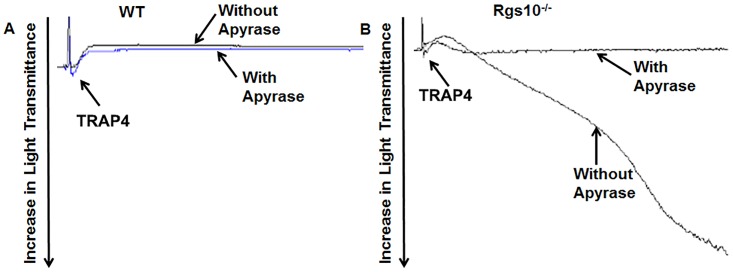
Aggregation in response to TRAP4 in the presence or absence of apyrase. Platelets from (A) WT or (B) *Rgs10*^***-/-***^ mice (2 x 10^8^/ml) were incubated with 0.5 units of apyrase for 3 minutes, then exposed to 40 μM TRAP4. Platelet aggregation was measured using constant stirring. Each experiment was repeated at least 3 times, with blood pooled from a group of eight mice.

Upon stimulation of platelets, granules undergo exocytosis thereby releasing coagulation factors and other substances. The enhanced aggregation of RGS10-deficient platelets mediated by TRAP4 could potentially be explained by secondary ADP signaling after secretion of ADP contained in α-granules [17]. To address this possibility, we incubated platelets from *Rgs10*^***-/-***^ or WT mice with the ADP-ATPase apyrase before treatment with TRAP4 (40 μM). Aggregation of RGS10-deficient platelets in response to TRAP4 was significantly inhibited after pre-incubation with apyrase, and aggregation became transient and reversible (Fig 3A and 3B). This finding suggests that the TRAP4-induced aggregation in platelets from *Rgs10*^***-/-***^ mice is heavily dependent upon secondary ADP secretion.

**Fig 4:**
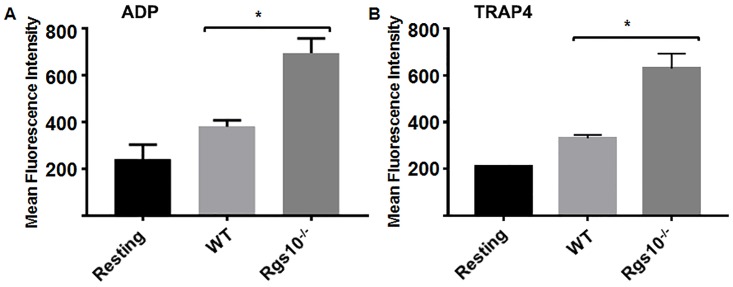
Deletion of RGS10 protein causes enhanced integrin αIIbβ3 activation in stimulated platelets. Platelets from *Rgs10*^***-/-***^ or WT mice (10^5^ platelets/100 μl) were stimulated with (A) 5 μM ADP and (B) 80 μM TRAP4, fixed, and labeled with JonA antibody. Samples were analyzed using a flow cytometer. Average mean fluorescence intensities shown. Experiment was conducted in duplicate, and was repeated at least 3 times, with blood pooled from a group of eight mice. (P<0.05 using Unpaired t-test).

Platelet aggregation occurs in part through a conformational change in the integrin αIIbβ3 on the platelet membrane, leading to integrin activation [18–20]. We measured cell surface expression of activated αIIbβ3 by flow cytometry. Although expression of activated αIIbβ3 was similar in resting platelets from WT and *Rgs10*^***-/-***^ mice, surface intensity of the active form of the integrin was significantly increased in RGS10-deficient platelets following stimulation with either ADP or TRAP4 compared to WT (Fig 4A and 4B). Taken together, these results indicate that platelets from *Rgs10*^***-/-***^ mice have an exaggerated aggregation response compared to the WT control, which occurs, at least in part, through an upregulation of α-granule secretion and through an integrin αIIbβ3-dependent mechanism.

**Fig 5:**
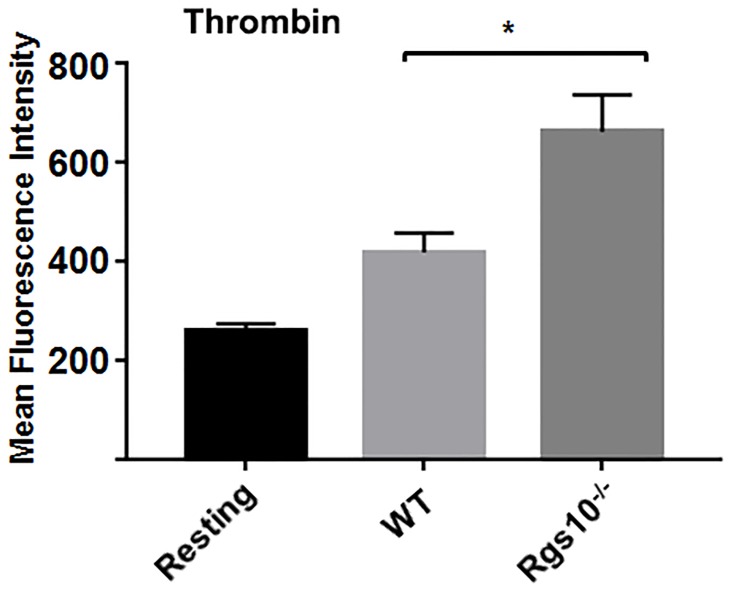
Deletion of RGS10 causes upregulation of α-granule secretion. Platelets from *Rgs10*^***-/-***^ or WT mice (10^5^ platelets/100 μl) were stimulated with 0.1 U/ml thrombin, fixed, and labeled with CD62P antibody. Samples were analyzed using a flow cytometer. Average mean fluorescence intensities shown. Experiment was conducted in duplicate, and repeated at least 3 times, with blood pooled from at least eight mice. (P<0.05 using Unpaired t test).

We also assessed whether RGS10 regulates α-granules release. This was accomplished by measuring the cell surface expression levels of the membrane-associated adhesion protein P-selectin by flow cytometry [21]. While resting platelets from WT or *Rgs10*^***-/-***^ mice exhibited comparable surface expression of P-selection, stimulation with thrombin induced a greater increase in P-selectin expression in platelets from *Rgs10*^***-/-***^ mice than in those from WT mice, suggesting exaggerated α-granule secretion (Fig 5).

### Deletion of RGS10 protein alters physiological hemostasis and thrombus formation

**Fig 6:**
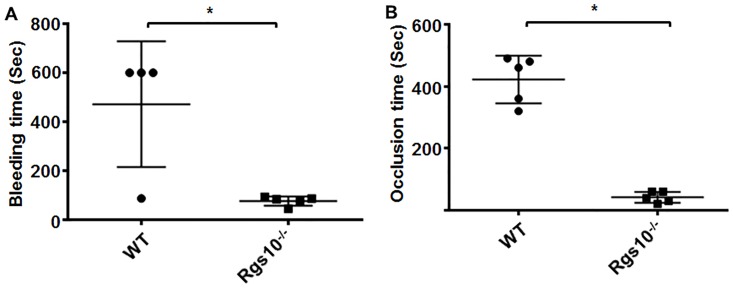
Deletion of RGS10 protein alters physiological hemostasis and development of thrombosis. (A) Bleeding times were measured in *Rgs10*^***-/-***^ (n = 5) or WT (n = 4) mice following venisection, as described in the “Methods” section. Each point represents the bleeding time of a single animal (P < 0.03 Mann-Whitney test). (B) Thrombosis was induced in *Rgs10*^***-/-***^ (n = 5) and WT (n = 5) mice using chemical injury (FeCl_3_), as described in the “Methods” section. Each point represents an occlusion time of a single animal (; P<0.0079 Mann-Whitney test).

To determine whether the *in vitro* hyper-responsiveness of platelets from *Rgs10*^***-/-***^ mice affects hemostasis *in vivo*, we measured bleeding times in live, anesthetized mice. Hemostasis was measured as the time needed for cessation of bleeding following tail transection with a scalpel. Using this technique, we observed significantly shortened bleeding times in *Rgs10*^***-/-***^ mice (~77 seconds) compared to wild-type littermates (~470 seconds) (Fig 6A). The wider spread of data seen from the WT mice can be explained by the heterogeneous nature of platelets, or differences between the strains of mice used. As this response depends almost completely on platelet quantities and activation, these results indicate that RGS10 negatively regulates the physiological hemostatic function of platelets *in vivo*.

Platelet hyperactivity is known to lead to thrombogenesis, which can lead to a blockage of blood flow. Because shortened bleeding times and augmented platelet activation suggested a hypercoagulable state, we hypothesized that *Rgs10*^***-/-***^ mice would be more prone to thrombosis.

To investigate whether RGS10 plays a role in regulating thrombogenesis, a chemically-induced vascular injury model was used to incite thrombus formation and vessel occlusion. FeCl_3_ was injected into a cannulized carotid artery in live, anesthetized mice, followed by measurement of the amount of time required for vessel occlusion (stoppage of blood flow detected by sonography) *Rgs10*^*-/-*^ mice were found to have dramatically shortened occlusion times, averaging approximately 40 seconds, when compared to the wild type littermates that on average had occlusion times of approximately 420 seconds (Fig 6B). These results demonstrate that RGS10 inhibits thrombus formation *in vivo*.

## Discussion

Regulators of G Protein Signaling (RGS) are well characterized in their ability to selectively target G-protein coupled receptors and regulate the downstream signaling cascade following their activation [4, 9, 22]. RGS10 has been shown to be expressed in hematopoietic cells—particularly peripheral blood mononuclear cells, immune cells, and platelets [23]. In previous work, RGS10 was found to bind to SHP-1 and negatively regulate platelet activation through a SPL dependent pathway [22], but whether or not it plays a direct role in platelet activation and physiology through its ability to deactivate specific GPCR pathways was not determined in this study. We investigated whether RGS10 plays a direct role in platelet function by stimulating purified platelets with GPCR ligands *in vitro* and by assessing platelet-dependent coagulation *in vivo*. Indeed, platelets from the *Rgs10*^***-/-***^ strain were hyper-responsive to specific agonists (ADP, TRAP4, thrombin, and U46619) compared to controls, as assessed by aggregation, α-granule secretion, and integrin αIIbβ3 activation. The lack of dose-dependence in the aggregation response may be due to an extremely hyperactive state of the RGS10- deficient platelets, at least with the agonists used and at the concentrations tested in our studies. The hyper-responsiveness of RGS10-deficient platelets conferred enhanced hemostasis (shortened bleeding time) and increased susceptibility to ischemia due to thrombosis in live mice. These findings suggest indispensible regulatory functions of RGS10 in platelet physiology. A limitation of our study is that because we used a strain containing a germline knockout of RGS10, we cannot formally exclude the effect of RGS10 deficiency on other cell types such as endothelium to the hypercoagulable state of these mice. However, endothelial expression of RGS10 has not been reported, and our *in vitro* studies demonstrating markedly enhanced platelet aggregation and activation argue strongly for a contribution of platelets to this phenotype.

Whether other individual RGS proteins have distinct or overlapping functions in platelets is not yet clear. Although we and others have demonstrated roles for R4 RGS family members RGS16 and RGS18 in platelet functions in mice, *Rgs2*^*-/-*^ mice were not more susceptible to platelet aggregation and thrombus formation at vascular injury locations relative to controls [24]. Similar to the phenotype of *Rgs10* gene-deleted mice, either RGS16- or RGS18-deficient platelets exhibited increased aggregation and secretion profiles compared to WT counterparts [11, 25]. However, in contrast to *Rgs18*^*-/-*^ mice, which have defective megakaryocyte maturation in bone marrow and impaired rebound of peripheral blood platelet quantities following acute thrombocytopenia [26], deletion of RGS10 did not appear to affect platelet development. Collectively, these studies indicate that functions of each individual RGS protein expressed in platelets may not be redundant.

In contrast to RGS2, 16, and 18, which belong to the R4 subfamily of RGS proteins, RGS10 is smaller in size and belongs to the R12 subfamily (consisting solely of RGS10 and RGS12). Although these RGS proteins have all been shown to accelerate the GTPase activity of Gαi, Gαq, and Gαz [2], RGS10 has structural features that suggest distinct physiological functions. For example, unlike the R4 subfamily members, which contain an amino-terminal amphipathic alpha helix mediating membrane localization, the cellular localization of RGS10 appears to be predominantly regulated by post-translational modifications including palmitoylation and phosphorylation [27–29]. In microglia, RGS10 may regulate the transcription factors NFκB and CREB through non-canonical mechanisms [23, 30]. With regards to the role of the other RGS proteins in platelet function, we have recently shown that RGS18 [25] deletion enhances platelet function and increases the risk of thrombosis. This finding suggests that RGS10 and 18 play similar/overlapping roles in platelet biology; this notion warrants investigation. We have also shown that RGS16 regulates chemokine-dependent agonist-induced signaling in platelets [11]. It is still to be determined whether RGS10 regulates chemokine-dependent agonist-induced signaling in platelets.

Our results clearly demonstrate that RGS10 negatively regulates platelet functions, which may eventually yield allosteric modifiers of specific GPCR pathways in these cells [31–33]. Hence, like RGS18, RGS10, is part of a braking system that limits platelet activation downstream of G proteins. For example, compounds enhancing RGS10 function may be effective for the treatment of hypercoagulable states, whereas RGS10 inhibitors could be developed for conditions associated with a bleeding diathesis [34]. Future studies of the full the spectrum of GPCR pathways regulated by RGS10 in platelets, as well as their signaling mechanisms, will be needed in order to develop such a therapeutic strategy.
